# Two age peaks in the incidence of chronic fatigue syndrome/myalgic encephalomyelitis: a population-based registry study from Norway 2008–2012

**DOI:** 10.1186/s12916-014-0167-5

**Published:** 2014-10-01

**Authors:** Inger Johanne Bakken, Kari Tveito, Nina Gunnes, Sara Ghaderi, Camilla Stoltenberg, Lill Trogstad, Siri Eldevik Håberg, Per Magnus

**Affiliations:** Norwegian Institute of Public Health, PO Box 4404, Nydalen, N-0403 Oslo, Norway; The Norwegian Medical Association, Oslo, Norway; Department of Global Public Health and Primary Care, University of Bergen, Bergen, Norway

**Keywords:** Chronic fatigue syndrome, Myalgic encephalomyelitis, Incidence rate, Sex, Age

## Abstract

**Background:**

The aim of the current study was to estimate sex- and age-specific incidence rates of chronic fatigue syndrome/myalgic encephalomyelitis (CFS/ME) using population-based registry data. CFS/ME is a debilitating condition with large impact on patients and their families. The etiology is unknown, and the distribution of the disease in the general population has not been well described.

**Methods:**

Cases of CFS/ME were identified in the Norwegian Patient Register (NPR) for the years 2008 to 2012. The NPR is nationwide and contains diagnoses assigned by specialist health care services (hospitals and outpatient clinics). We estimated sex- and age-specific incidence rates by dividing the number of new cases of CFS/ME in each category by the number of person years at risk. Incidence rate ratios were estimated by Poisson regression with sex, age categories, and year of diagnosis as covariates.

**Results:**

A total of 5,809 patients were registered with CFS/ME during 2008 to 2012. The overall incidence rate was 25.8 per 100,000 person years (95% confidence interval (CI): 25.2 to 26.5). The female to male incidence rate ratio of CFS/ME was 3.2 (95% CI: 3.0 to 3.4). The incidence rate varied strongly with age for both sexes, with a first peak in the age group 10 to 19 years and a second peak in the age group 30 to 39 years.

**Conclusions:**

Early etiological clues can sometimes be gained from examination of disease patterns. The strong female preponderance and the two age peaks suggest that sex- and age-specific factors may modulate the risk of CFS/ME.

## Background

Chronic fatigue syndrome (CFS), or myalgic encephalomyelitis (ME), is a debilitating, medically unexplained condition [1]. The terms CFS and ME are often used interchangeably, and Norwegian health authorities recommend using the combined term CFS/ME [2].

CFS/ME is an unspecific condition for which it has been difficult to establish objective medical criteria, and the CFS/ME diagnosis has been debated in the medical community for many years [3]. Symptoms may fluctuate and vary in intensity within and among patients, but persistent or relapsing fatigue is always present [4]. Functional status and wellbeing are often strongly affected [5].

While the etiology of CFS/ME remains largely unknown, several trigger mechanisms have been proposed, including infections, stress and trauma [1]. A sudden increase in CFS/ME was reported following a large waterborne outbreak of giardiasis in Bergen, Norway, in 2004 [6]. Autoimmune etiology has also been suggested, based on the observation that B-lymphocyte depletion with the monoclonal anti-CD20 antibody rituximab was associated with transient symptom improvement in some patients with CFS/ME [7].

Due to terminological variations and diagnostic inconsistencies, it is difficult to assess the prevalence and incidence rate of CFS/ME in a population. Overall prevalence estimates vary from 0.1% to 2.5%, depending on the criteria applied [1,8].

The aim of the current study was to estimate sex- and age-specific incidence rates of CFS/ME in Norway during a five-year period, using data from a nationwide registry containing diagnoses assigned by Norwegian specialist health care services (hospitals and outpatient clinics).

## Methods

The use of national data and data linkage procedures were approved by the Regional Committee for Medical and Health Research Ethics, South-East Norway. The ethical approval permitted the use of national level data. The research group did not at any time point have access to names or personal identification numbers, and all data storage and handling have been carried out within strict standards to ensure data privacy and protection.

The study population was the complete Norwegian population five years old and older as of 1 January 2008, registered in the Central Population Registry. For each individual we had access to information on vital status (dates for emigration or death) for the entire study period, 2008 to 2012. All individuals were assigned a study allocation number based on the 11-digit personal identification number unique to all Norwegian citizens and migrants with legal residence in Norway.

Information on CFS/ME was obtained from the Norwegian Patient Register (NPR), which is a database containing data from all Norwegian hospitals and outpatient clinics. For each hospitalization or outpatient visit, reporting of data to the NPR is mandatory and is linked to the reimbursement system. Discharge diagnoses are reported as International Classification of Disease, version 10 (ICD-10) codes. Personal identification numbers have been reported to the NPR from 2008 onwards, making tracking of subjects possible for research purposes. Reporting of the personal identification number has been nearly complete from the start [9]. The personal identification number is stored in encrypted form in the NPR.

Cases of CFS/ME were all in- and outpatients in Norwegian hospitals who were registered with the ICD-10 code G93.3 (‘postviral fatigue syndrome/benign myalgic encephalomyelitis’). Data from mental health care facilities were not included. In addition to the ICD-code, the NPR provided information about sex, year of birth, dates for hospitalizations and outpatient visits, and a study allocation number for data linkage. The first registered G93.3 episode for each patient was used in the analyses.

Age was calculated as the age in 2008 by subtracting year of birth from calendar year. We estimated sex- and age-specific incidence rates by dividing the number of new cases of CFS/ME in each sex/age category by the total number of person years at risk in the same category. Time at risk for each individual was calculated by using the information on vital status. Age was categorized in five-year intervals (5 to 9, 10 to 14, …, 55+). We estimated the incidence rate ratio of CFS/ME by applying Poisson regression with sex, age categories, and year of diagnosis as covariates and compared estimated incidence rates in sex and age categories.

The Stata software package, Version 11.2 (StataCorp. 2009. *Stata Statistical Software: Release 11*. College Station, TX, USA: StataCorp LP) was used for data analysis.

## Results

**Figure 1:**
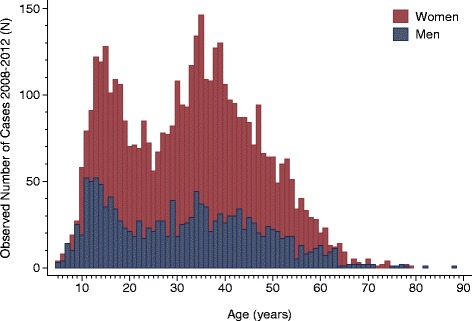
**Observed number of chronic fatigue syndrome/myalgic encephalomyelitis (CFS/ME) cases by sex and one-year age groups (age in 2008).** Data from the Norwegian Patient Register 2008 to 2012.

During the years 2008 to 2012, 5,809 patients (75.4% women) five years old or older in 2008 were registered with CFS/ME. Figure 1 shows the number of cases in one-year age intervals for women and men separately. For both sexes, two distinct peaks in the number of cases were observed, the first in the age group 10 to 19 years and the second in the age group 30 to 39 years.

Table 1 shows the incidence rate of CFS/ME by year, sex and age category. The incidence rate was fairly stable over the years of follow-up. The overall incidence rate per 100,000 person years was 39.4 (95% confidence interval (CI): 38.2 to 40.6) for women, while the corresponding figure was 12.9 (95% CI: 12.3 to 13.6) for men. This gives an incidence rate ratio of 3.2 (95% CI: 3.0 to 3.4) for women compared to men. The incidence rate was highest in the age groups 10 to 14, 15 to 19, 30 to 34 and 35 to 39 years.

**Table 1:** **Incidence rates and incidence rate ratios of CFS/ME in the Norwegian population according to year, sex and age in 2008 (categorized), 2008 to 2012**

**Variable**	**Number of cases**	**Number of person years**	**Incidence rate per 100,000 person years (95% CI)**	**Incidence rate ratio (95% CI)**
Year				
2008	1,172	4,494,062	26.1 (24.6 to 27.6)	1.0 (ref.)
2009	1,113	4,491,822	24.8 (23.3 to 26.3)	0.9 (0.9 to 1.0)
2010	1,104	4,449,683	24.8 (23.4 to 26.3)	0.9 (0.9 to 1.0)
2011	1,222	4,395,325	27.8 (26.3 to 29.4)	1.0 (1.0 to 1.1)
2012	1,198	4,342,818	27.6 (26.1 to 29.2)	0.9 (0.9 to 1.1)
Sex				
Male	1,430	11,050,966	12.9 (12.3 to 13.6)	1.0 (ref.)
Female	4,379	11,122,744	39.4 (38.2 to 40.6)	3.2 (3.0 to 3.4)
Age category (years)				
5 to 9	121	1,497,289	8.1 (6.7 to 9.7)	1.3 (1.1 to 1.6)
10 to 14	690	1,578,106	43.7 (40.5 to 47.1)	7.1 (6.3 to 8.1)
15 to 19	689	1,598,802	43.1 (39.9 to 46.4)	7.0 (6.2 to 8.0)
20 to 24	473	1,481,322	31.9 (29.1 to 34.9)	5.2 (4.5 to 5.9)
25 to 29	492	1,523,923	32.3 (29.5 to 35.3)	5.2 (4.6 to 6.0)
30 to 34	688	1,603,520	42.9 (39.6 to 46.2)	7.0 (6.1 to 7.9)
35 to 39	771	1,809,970	42.6 (39.6 to 45.7)	6.9 (6.1 to 7.8)
40 to 44	614	1,797,270	34.2 (31.5 to 37.0)	5.6 (4.9 to 6.3)
45 to 49	496	1,636,483	30.3 (27.7 to 33.1)	4.9 (4.3 to 5.6)
50 to 54	383	1,564,478	24.5 (22.1 to 27.1)	4.0 (3.4 to 4.6)
55+	392	6,082,547	6.2 (5.6 to 6.9)	1.0 (ref.)

CFS/ME, chronic fatigue syndrome/myalgic encephalomyelitis; CI, confidence interval.

**Figure 2:**
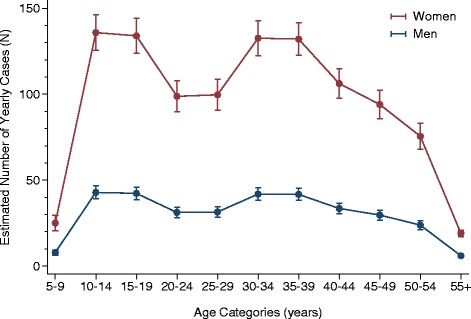
**Number of yearly cases of CFS/ME by sex and five-year age groups estimated by Poisson regression analyses.** CFS/ME, chronic fatigue syndrome/myalgic encephalomyelitis.

Figure 2 shows the number of cases per year by age category for men and women separately, estimated from the Poisson regression model. The non-overlapping CIs support the existence of two age peaks in the distribution indicated by the raw data (Figure 1).

**Figure 3:**
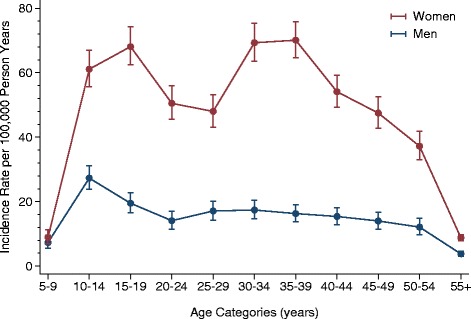
**Estimated incidence rates of CFS/ME per 100,000 person years by sex and five-year age groups.** CFS/ME, chronic fatigue syndrome/myalgic encephalomyelitis.

Figure 3 shows the estimated incidence rate per 100,000 person years for men and women separately. After adjustment for population figures, the pattern with two age peaks is clear for women, whereas a second peak is not evident for men.

Repeating the analyses including a ‘wash-out’ period of two years (that is, excluding data for all patients first registered in 2008 and 2009) gave similar sex and age patterns as those described here (results not shown). Norway is divided into four health regions, with most people living in the South-East region. Although some variations were observed, the pattern with a strong preponderance of women and higher risk of CFS/ME in the age groups 10 to 19 years and 30 to 39 years also persisted when performing the analyses for each health region separately (results not shown).

## Discussion

This is the largest study to date of CFS/ME, including 5,809 patients and using national population-based registry data. To our knowledge, this is also the first study to investigate the distribution of CFS/ME by sex *and* age in a population. The main new finding is the distinct age peaks in the incidence of CFS/ME, with a first peak in the number of cases in the age group 10 to 19 years and a second peak in the age group 30 to 39 years. Although a higher prevalence in women than in men has been a consistent finding in previous studies [1], most epidemiological studies so far have been too small to report age distributions [10-15] or have only reported the age distribution for women and men combined [16,17].

The strength of the current study is the access to individual-level hospital data from an entire country, which could be linked to high-quality population data by means of personal identification numbers. In Norway, access to specialist health care services requires referral from a general practitioner, and the cases included in the NPR probably represent the more severe and established cases of CFS/ME. The Norwegian health care system is financed through governmental funding. All hospitalizations are free of charge, while outpatients 16 years old or older are charged a minor fee. Thus, economic status should have little influence on patterns of seeking health care.

It is reasonable to assume that the more severe cases of CFS/ME are referred to specialist health care services, and that this is also the case for young patients and people with sudden onset of symptoms. Even so, the estimated incidence rates in the current study may differ from the true population incidence. Since our study is based on diagnoses reported from specialist health care services, we have calculated the incidence of being diagnosed with the condition and not the incidence of the condition as such. Also, some patients regarded as incident cases in the current study may have been diagnosed the first time before the start of follow-up. Such cases will contribute to an overestimation of the true incidence rate. On the other hand, patients with less severe CFS/ME are probably in many cases only diagnosed in the primary health care system, a mechanism that leads to underestimation of the incidence rate. However, it seems unlikely that such factors have severely biased the incidence rate ratio of women versus men or the incidence rate ratios of the different age categories seen in our study.

The main weakness of this study is the possible variation in the use of ICD-10 code G93.3 for CFS/ME due to the inherent heterogeneity of the disease. We did not have access to information from medical records and could not investigate the diagnostic criteria applied in individual patients. According to the recommendations from the Norwegian Directorate of Health, children with suspected CFS/ME should have the diagnosis confirmed by a pediatrician [2]. Thus, children receiving the diagnosis of CFS/ME are routinely referred to their local hospital for the diagnostic work-up. Complicated cases may be further referred to regional university hospitals. After the diagnosis of CFS/ME is made, patients are typically followed up in primary care. Adults may receive a diagnosis of CFS/ME after evaluation by a general practitioner. However, since the diagnosis requires that other possible diagnoses are ruled out, most patients have most likely also been seen by a specialist at a local hospital during the diagnostic work-up. Patients may also be referred to one of the four university hospitals which have specialized units for diagnosing CFS/ME or to the national CFS/ME-center at Oslo University Hospital. Follow-up after diagnosis is the responsibility of general practitioners.

The Norwegian Directorate of Health has stated that ICD-10 code G93.3 is to be used for this disorder [2]. However, cases in the present study come from a large number of different hospitals and the criteria used to diagnose CFS/ME might have varied. In a recent study, most Norwegian hospitals reported that they had used either the Canadian 2003 criteria [18] or the CDC 1994 criteria [4] when diagnosing CFS/ME in adults [19]. Pediatricians generally reported they follow the guidelines of the Norwegian Pediatric Association [20], which formally refer to the CDC 1994 criteria, but also recommend using a practical, clinical definition of CFS/ME in children with otherwise unexplained severe fatigue of more than three months duration. We cannot exclude the possibility that the relatively high incidence in children and adolescents may be partly due to the use of a less strict case definition of CFS/ME in young people. However, we find it unlikely that this potential over-reporting is substantial, as pediatricians probably are restrictive in using this diagnosis.

Some patients fulfilling the criteria for a diagnosis of CFS/ME may be missed in the current study due to the possible use of ICD-10 codes for other conditions with overlapping symptoms, such as neurasthenia, burn-out syndrome, malaise and fatigue, or fibromyalgia. However, while a diagnosis of G93.3 gives the right to social security benefits, such as sickness benefit or disability pension, this is most often not the case for, for example, neurasthenia, which includes many of the same symptoms but to a milder degree [2]. According to the guide from the Norwegian Directorate of Health, the code for neurasthenia should only be used when CFS/ME has been ruled out [2]. The ICD-10 guidelines also state that F48.0 (‘neurasthenia’) excludes G93.3 [21]. Thus, we regard G93.3 as the ICD-10 code most closely related to CFS/ME.

The incidence rates reported here are somewhat higher than the rates reported from the UK for the time period 1990 to 2001 [16], while the female to male ratios are comparable. Although the prognosis for an improvement in symptoms of CFS/ME is fairly good, full recovery seems rare [22]. The prognosis for children and adolescents seems to be better than for adults [23,24]. Due to the long duration of the disorder, our study estimating incidence rates is not directly comparable with previous prevalence surveys. However, the large sex difference reported here is in line with results from previous reports [1,8]. The previous prevalence studies are small with respect to the number of cases, and the scarce data available have usually limited analyses of sex and age distributions. For instance, from the UK, an overall prevalence of 0.30% for women and 0.09% for men and an estimated minimum yearly incidence at 15/100,000 has been reported [14]. This study was based on direct questioning of general practitioners combined with electronic database searches that covered 143,000 individuals 18- to 64-years old and included data from 122 cases only. Even stronger sex differences were reported from a US study based on medical record review following electronic searches in databases [15]. This latter study covered both primary and specialist health care and reported a prevalence of 0.12% for women and 0.02% for men, based on data from only 76 identified cases in total. Survey-based approaches have also been utilized in several studies. In a telephone random-digit sampling study from the US, 43 cases were found among 56,146 participants [17]. The weighted prevalence estimates in that study were 0.37% for women and 0.08% for men. The study also reports the age distribution, but not for women and men separately. In a survey from Japan carried out among 1,430 participants in a health check-up program [11], the prevalence was estimated at 1.0%, based on 8 male and 6 female cases.

Although results from some questionnaire studies related to fatigue in general have been published from Denmark [25], Iceland [13] and Norway [26], only the Icelandic study attempts to report the prevalence of CFS/ME. In the study from Iceland, the sex distribution was similar to that in the current study, but the prevalence was higher (3.0% for women and 1.1% for men), based on a total of only 54 cases. In the Danish study, women had higher fatigue scores than men in a sample of 1,082 individuals responding to a questionnaire, and the variation in scores was also higher among women than among men, but CFS/ME cases were not defined [25]. The previous Norwegian study showed that the prevalence of general fatigue was high, with a slightly higher proportion of women than men with high scores on a fatigue questionnaire in a random population sample (N = 2,323) [26].

We observed a distinct age pattern in the incidence rate of CFS/ME, with the number of cases peaking in the age groups 10 to 19 years and 30 to 39 years. This pattern may suggest that development of CFS/ME might be caused by different etiological factors in different age groups, but could also be explained by increased vulnerability in these age groups. The female preponderance of CFS/ME indicates that sex hormones may play a part in the development of the condition. Puberty and the years after puberty are well-known vulnerable periods for the debut of several diseases, including autoimmune and psychiatric disorders [27]. Even though most women have not yet reached menopause in their late thirties, many women have given birth by this age. In pregnancy and postpartum periods, the risk of a wide range of conditions is increased due to rapid hormonal changes. For instance, migraine is a predominantly female disorder [28]. Menarche, menstruation, childbirth and menopause influence the frequency of migraine, and sex steroids are considered to play an important role [29]. Migraine is also highly prevalent in patients with CFS/ME [30]. Sex steroids cross the blood–brain barrier by passive diffusion and are also produced within the central nervous system [31]. These neurosteroids modulate brain networks and alter brain excitability [32]. Estrogen and progesterone are known to influence a range of neurotransmitters including serotonin, norepinephrine, dopamine and endorphins. Estrogen facilitates the glutamatergic system, potentially enhancing neuronal excitability, whereas progesterone activates GABAergic tone and suppresses neuronal reactivity. Changes in neurotransmitter systems can affect, for example, pain-processing networks, modulation of sensory input and cognition. Such mechanisms have been suggested to play a role in fibromyalgia [33] and may possibly also come into play in the development of CFS/ME.

Far more women than men are affected by multiple sclerosis (MS), and it has been suggested that the strong phenomenological, neurobehavioral and neuroimmune similarities between MS and CFS/ME indicate that CFS/ME is a neuroimmune disorder [34].

From the current results, one might hypothesize that hormonal changes increase the risk of CFS/ME in women. The pattern may also be compatible with the hypothesis of an infectious trigger for the condition. The age distribution observed in our study may be explained by primary exposure to an infectious agent in adolescents (first age peak) and subsequent reactivation of latent infection (second age peak). It has been shown that reactivation of latent (viral) infections may be triggered by stressful events, chronic stress or pregnancy [35].

## Conclusions

In summary, in this large national study, we found that the CFS/ME risk is strongly dependent on sex and age. The distinctive sex and age patterns might be helpful when exploring potential causal mechanisms. Also, our findings indicate that clinicians should have a heightened awareness of the possibility of CFS/ME, especially in women in certain age groups presenting with symptoms of fatigue.

## Abbreviations

CFS: chronic fatigue syndrome
CFS/ME: chronic fatigue syndrome/myalgic encephalomyelitis
CI: confidence interval
ICD-10: International Classification of Disease, version 10
ME: myalgic encephalomyelitis
MS: multiple sclerosis
NPR: Norwegian Patient Register
